# Supplementary material for: A Two-Step Approach to Overcoming Data Imbalance in the Development of an Electrocardiography Data Quality Assessment Algorithm: A Real-World Data Challenge
Source: Biomimetics (Basel). 2023 Mar 13;8(1):119. doi: 10.3390/biomimetics8010119 (PMC10046279; doi:10.3390/biomimetics8010119)
Supplement: Supplementary file 1 [file biomimetics-08-00119-s001.zip › biomimetics-2251076-supplementary.pdf]

Supplementary Material for the Manuscript Entitled

A Two-Step Approach to Overcoming Data Imbalance in the Development of an Electrocardiography Data Quality Assessment Algorithm: A Real-World Data Challenge

Hyun Joo Kim [1], S. Jayakumar Venkat [2], Hyoung Woo Chang [2,*], Yang Hyun Cho [3], Jee Yang Lee [2] and Kyunghee Koo [2]

[1] Department of Anesthesiology and Pain Medicine, Anesthesia and Pain Research Institute, Severance Hospital, Yonsei University College of Medicine, Seoul 03722, Republic of Korea
[2] Department of Thoracic and Cardiovascular Surgery, Seoul National University Bundang Hospital, Seoul National University College of Medicine, Gyeonggi-do, Seongnam-si 13620, Republic of Korea
[3] Department of Thoracic and Cardiovascular Surgery, Samsung Medical Center, Sungkyunkwan University College of Medicine, Seoul 06351, Republic of Korea
* Correspondence: chang.hyoungwoo@gmail.com; Tel.: +82-31-787-7151; Fax: +82-31-787-4050

Table of Contents

Section S1. Examples of each quality class

Figure S1a. *acceptable* class segments

Figure S1b. *unacceptable* class segments

Figure S1c. *uncertain* class segments

Section S2. Fundamentals of feature extraction

Section S3. Feature extraction algorithms

Figure S2a. Algorithms to extract time domain features (pseudocode)

Figure S2b. Algorithms to extract frequency domain features (pseudocode)

Section S4. Loss graphs of the 2D CNN approaches

Figure S3a. Loss graphs of the one-step three-class 2D CNN (*acceptable*, *unacceptable* and *uncertain*)

Figure S3b. Loss graphs of the first-step binary 2D CNN classifier of the two-step approach (*acceptable* vs. *unacceptable* + *uncertain*)

Figure S3c. Loss graphs of the second-step binary 2D CNN classifier of the two-step approach (*unacceptable* vs. *uncertain*)

Section S5. Confusion matrices of the raw test data for the four approaches: 3 × 3 and 2 × 2

Figure S4a. The one-step three-class random forest approach

Figure S4b. The one-step three-class 2D CNN approach

Figure S4c. The two-step binary sequential random forest approach

Figure S4d. The two-step binary sequential 2D CNN approach

Section S6. Source Codes

File S1a. Dataingestor.py (preprocess)

File S1b. FeatureExtractor.py (feature extraction codes for random forest model)

File S1c. Codes for training random forest model for differentiation between *acceptable* and *unacceptable*

File S1d. Codes for training of 2D CNN (binary classifier for *unacceptable* vs *uncertain*)

Section S1. Examples of each quality class

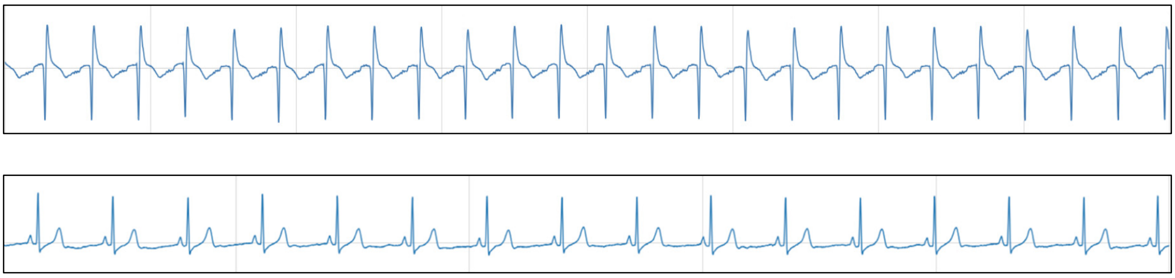

Figure S1a. *acceptable* class segments

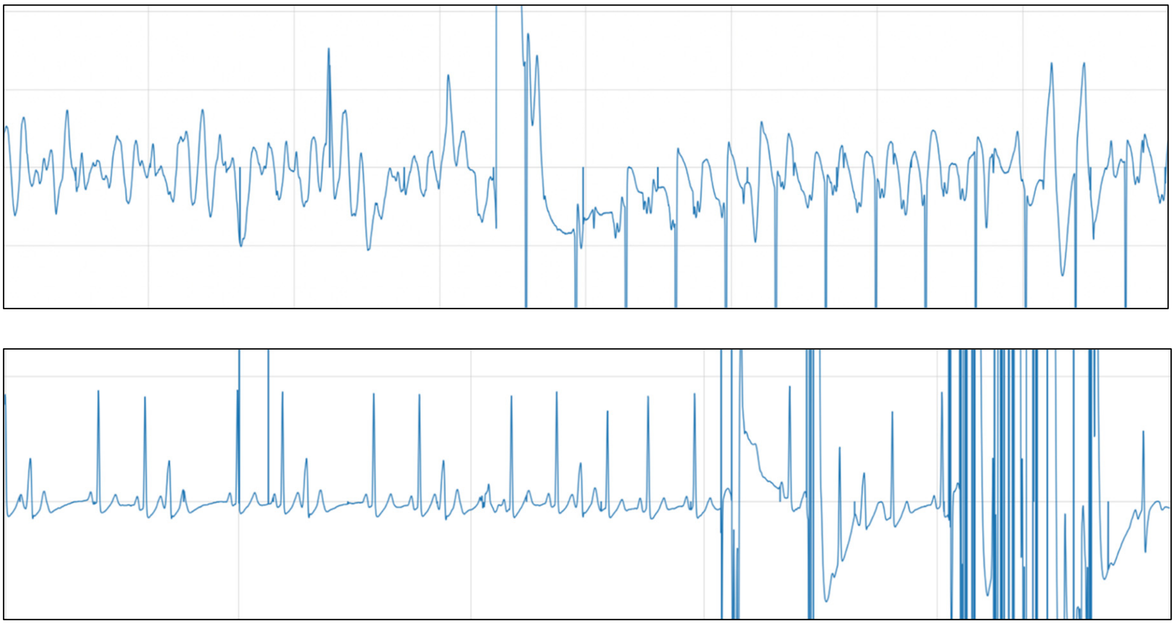

Figure S1b. *unacceptable* class segments

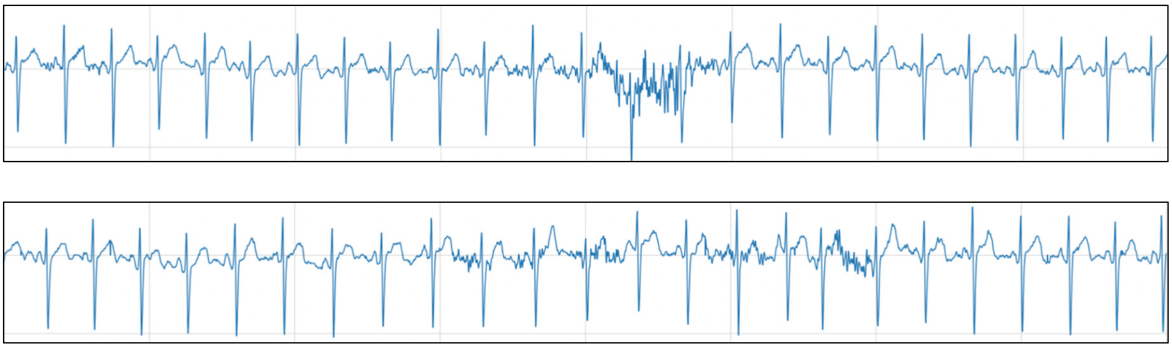

Figure S1c. *uncertain* class segments

Section S2. Fundamentals of feature extraction

1) Slope—The slope of the rising edge (Q-R) of the ECG signal is calculated using the peaks of the signal and the peaks of the negated signal. The slope calculation is shown in Section 3 (lines 35-40).

2) Peak amplitude—Peak amplitudes are obtained using the *heartpy* peak detection routine from the filtered signal. The same process is used to obtain peaks from the negated signal.

3) Z score—The Z score is computed for the peak amplitudes obtained from the filtered signal. It is also computed for the peak amplitudes obtained from the negated signal.

4) Signal-to-noise ratio—The SNR is computed as the ratio of the mean to the standard deviation of the signal.

5) Auto Correlation—The *statsmodel* python package is used to compute the auto correlation for every 1000 samples of the signal. The same process is applied to the negated signal. L*ags* is set to 3.

6) Cross correlation—To extract the amount of similarity between 2 time series, the *scipy.signal.correlate* package is used to compute cross correlations between 2 nonoverlapping subsegments with 1000 samples from the signal. The same process is also applied to the negated signal.

7) Skew—The skew is computed from the filtered signal using the *scipy.stats* package.

8) Kurtosis—Kurtosis is computed from the signal as well as the negated signal using the *scipy.stats* package.

9) Dynamic Time Warping—The *dtaidistance* package is used to compute the dynamic time warped distance between 2 nonoverlapping subsegments with 1000 samples from the signal. The same process is also applied to the negated signal.

10) Hurst exponent—The Hurst exponent shows the degree of persistence or anti-persistence of a time series. The Hurst Python package is used to compute the Hurst exponent of the signal. The Hurst exponent is also calculated for the negated signal.

11) maxpower—From every 1000 samples of the signal, the maximum power is obtained after a Fourier transformation. The same process is applied to the negated signal. The MaxPower computation is

shown in Figure 2.

12) maxpowerdiff—Power values obtained from every 1000 samples of the signal are sorted in descending order. The difference between the first 5 power values is obtained to capture information in the power signatures of acceptable, unacceptable, and uncertain signals. This is shown in Section S3 Figure S2b.

13) meanfreqdiff—Frequency windows with the maximum power in every 1000 samples are obtained. The meanfreqdiff computation is shown in Section S3 Figure S2b.

14) Short-time Fourier transforms (STFT) are computed using *scipy.signal.stft* to convert the signal into the time-frequency domain.

15) Spectral Entropy—The *Neurokit2* python package is used to compute spectral entropy. The signal's normalized power distribution in the frequency domain is treated as a probability distribution, and its Shannon entropy is computed. Similarly, spectral entropy is computed for the negated signal.

16) First derivative—The first derivative of the ECG signal is a popular feature to detect the rate of change. Sharp peaks and notches result in large fluctuations in the derivative.

17) Second derivative—The second derivative of the ECG signal is also taken, although applications of this derivative in ECG analysis or classification are less than those of the first derivative.

Section S3. Feature extraction algorithms

Figure S2a. Algorithms to extract time domain features (pseudocode)

```
N = 5000
sf = 250
x = 1000
i = 0
j = 1000
signal_filtered ← filter(signal, ”bandpass”, [0.5, 8])
negsignal_filtered ← filter(−signal, ”bandpass”, [0.5, 8])
peaktimes, peakamplitudes ← getpeaks(signal_filtered)
peaktimes_neg, peakamplitudes_neg ← getpeaktimes(negsignal_filtered)
zscore_time ← zscore(peaktimes)
zscore_negtime ← zscore(peaktimes_neg)
zscore ← zscore(peakamplitudes)
zscore_neg ← zscore(peakamplitudes_neg)
meanpeakamplitudes_total ← []
maxpeakamplitudes_total ← []
minpeakamplitudes_total ← []
meanpeakintervals_total ← []
maxpeakintervals_total ← []
minpeakintervals_total ← []
meanpeakamplitudes_negtotal ← []
maxpeakamplitudes_negtotal ← []
minpeakamplitudes_negtotal ← []
meanpeakintervals_negtotal ← []
maxpeakintervals_negtotal ← []
minpeakintervals_negtotal ← []
slopes ← []
f' ← gradient(signal_filtered)
f'' ← gradient(f')
acf ← autocorr(signal_filtered)
acf_neg ← autocorr(negsignal_filtered)
skew ← skew(signal_filtered)
skew_neg ← skew(negsignal_filtered)
kurtosis ← kurtosis(signal_filtered)
kurtosis_neg ← kurtosis(negsignal_filtered)
snr ← mean(signal_filtered)/stddev(signal_filtered)
snr_neg ← mean(negsignal_filtered)/stddev(negsignal_filtered)
he ← HurstExponent(signal_filtered)
he_neg ← HurstExponent(negsignal_filtered)
crosscorr ← []
dtw ← []
skewchange ← []
skewchange_neg ← []
kurtochange ← []
kurtohange_neg ← []
for peaktime in peaktimes do
    y ← getamplitudes(peaktime)
    x ← max(peaktimes_neg[peaktimes_neg < peaktime])
    y2 ← peakamplitudes_neg(x)
    slopes.insert((y − y2)/(peaktime − x))
end for

for i<N do

meanpeakamplitudes_total.insert(mean(peakamplitudes (i:i+x))

maxpeakamplitudes_total.insert(max(peakamplitudes (i:i+x)))
minpeakamplitudes_total.insert(min(peakamplitudes (i:i+x)))
meanpeakamplitudes_negtotal.insert(mean(peakamplitudes_neg(i:i+x)) )
maxpeakamplitudes_negtotal.insert(max(peakamplitudes_neg(i:i+x))) )
```

```
minpeakamplitudes_negtotal.insert(min(peakamplitudes_neg(i:i+x)) )
meanpeakintervals.insert(mean(diff(peaktimes(i:i+x))) )
maxpeakintervals.insert(max(diff(peaktimes(i:i+x))) )
minpeakintervals.insert(min(diff(peaktimes(i:i+x))) )
meanpeakintervals_negtotal.insert(mean(diff(peaktimes_neg(i:i+x))) )
maxpeakintervals_negtotalnegtotal.insert(max(diff(peaktimes_neg(i:i+x))) )
minpeakintervals_negtotal.insert(min(diff(peaktimes_neg(i:i+x))) )
amplitudeskew.insert(skew(peakamplitudes(i:i+x)) )
amplitudeskew_neg.insert(skew(peakamplitudes_neg(i:i+x)) )
amplitudekurtosis.insert(kurtosis(peakamplitudes(i:i+x)) )
amplitudekurtosis_neg.insert(kurtosis(peakamplitudes_neg(i:i+x)) )
timeskew.insert(skew(peaktimes(i:i+x)) )
timeskew_neg.insert(skew(peaktimes_neg(i:i+x)) )
timekurtosis.insert(kurtosis(peaktimes(i:i+x)) )
timekurtosis_neg.insert(kurtosis(peaktimes_neg(i:i+x)) )
i = i + x
end for
i=0
for i<N do
    for j+1000<5000 do
        crosscorr.insert(crosscorr(signal[i : j]_filtered, signal[j : j +1000]_filtered))
        crosscorr_neg.insert(crosscorr(negsignal[i : j]_filtered, negsignal[j : j + 1000]_filtered))
        dtw.insert(dynamicTimeWarping(signal[i : j]_filtered, signal[j : j+1000]_filtered))
        dtw_neg.insert(dynamicTimeWarping(negsignal[i :j]_filtered, negsignal[j : j + 1000]_filtered))
    end for
    i=j
    j=j+1000
end for
features_pre ← [meanpeakamplitudes_total, maxpeakamplitudes_total, minpeakamplitudes_total, meanpeakamplitudes_negtotal,
    maxpeakamplitudes_negtotal, minpeakamplitudes_negtotal, meanpeakintervals_total, maxpeakintervals_total, minpeakintervals_total,
    meanpeakintervals_negtotal, maxpeakintervals_negtotal, minpeakintervals_negtotal, zscore, zscore_neg, zscore_time, zscore_negtime,
    slopes, crosscorr, dtw, crosscorr_neg, dtw_neg, f', f'', acf, acf_neg, snr, snr_neg, amplitudeskew, amplitudeskew_neg,
    amplitudekurtosis, amplitudekurtosis_neg, timeskew, timeskew_neg, timekurtosis, timekurtosis_neg]
for feature in features_pre do
features.insert(mean(feature))
features.insert(std(feature))
end for
features.insert(max(zscore))
features.insert(min(zscore_neg))
features.insert(he)
features.insert(he_neg)
```

Figure S2b. Algorithms to extract frequency domain features (pseudocode)

```
N = 5000
sf = 250
x = 1000
i = 0
freq_window ← [1, 2, ...8]
meanfreqdiff ← []
meanpowerdiff ← []
maxpower ← []
stdpower ← []
stft_neg ← abs(STFT(negsignal_filtered))
stft ← abs(STFT(signal_filtered))
se_neg ← SpectralEntropy(negsignal_filtered)
se ← SpectralEntropy(signal_filtered)
for i<N do
      power_sample ← abs(FFT(signal_i:i+x))
      maxpower.insert(max(power_sample)
      maxfreqbywindow ← []
      for f in freq_window do
            r ← [f − 1, ..., f − .2, f − .1, f]
            for s in r do
                  if power_sample(s) == max(power_sample) : maxfreqbywindow.insert(s)
            end for
      end for
      meanfreqdiff.insert(mean(diff(maxfreqbywindow)))
      meanpowerdiff.insert(mean(diff(sort_desc(maxpower)[:5])))
      i ← i + x
end for
features.insert(stddev(meanfreqdiff))
features.insert(stddev(meanpowerdiff))
features.insert(mean(maxpower))
features.insert(stddev(maxpower))
features.insert(mean(stft))
features.insert(std(stft))
features.insert(mean(stft_neg))
features.insert(std(stft_neg))
features.insert(mean(se_neg))
features.insert(std(se_neg))
features.insert(mean(se))
features.insert(std(se))
```

Section S4. Loss graphs of the 2D CNN approaches

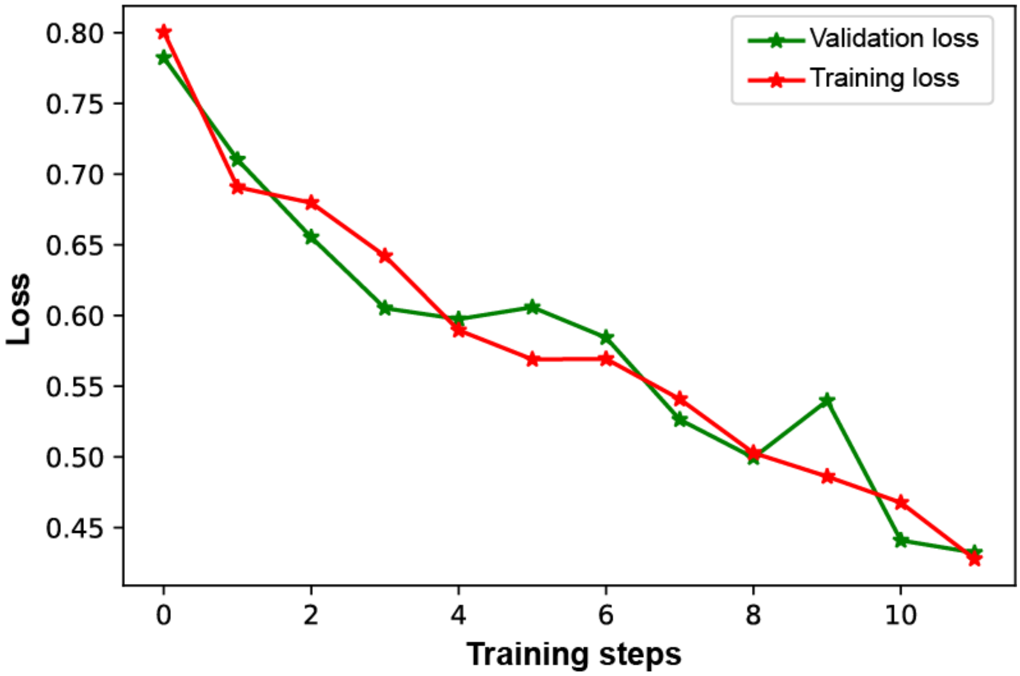

Figure S3a. Loss graphs of the one-step three-class 2D CNN (*acceptable*, *unacceptable*, and *uncertain*)

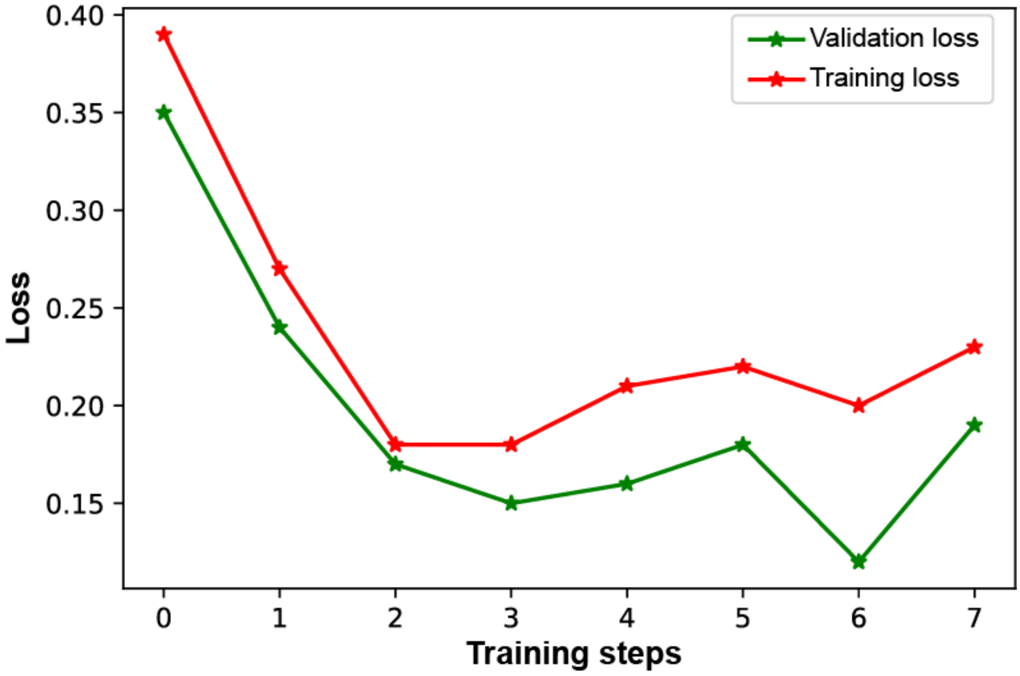

Figure S3b. Loss graphs of the first-step binary 2D CNN classifier in the two-step approach (*acceptable* vs. the others (*unacceptable* + *uncertain*))

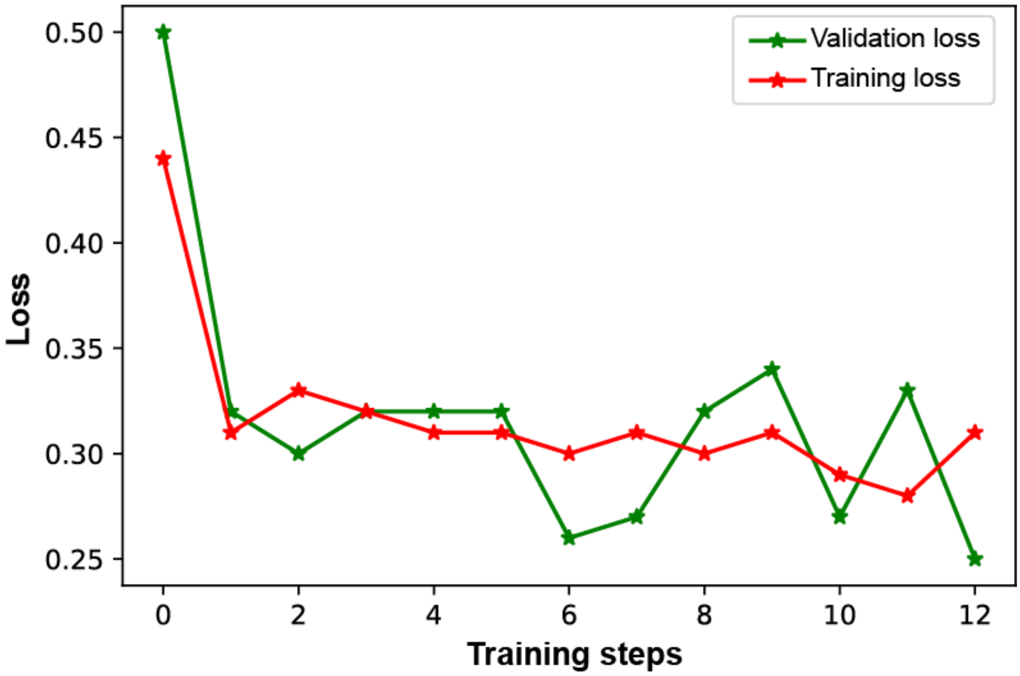

Figure S3c. Loss graphs of the second-step binary 2D CNN classifier in the two-step approach (*unacceptable* vs. *uncertain*)

Section S5. Confusion matrices of the raw test data for the four approaches: 3×3 and 2×2

True unacceptable	505	19	187
True acceptable	332	6,222	1,581
True uncertain	233	86	614
	Predicted unacceptable	Predicted acceptable	Predicted uncertain

True unacceptable	505	206
True acceptable +uncertain	565	8,503
	Predicted unacceptable	Predicted acceptable +uncertain

Figure S4a. The one-step three-class random forest approach

True unacceptable	470	32	209
True acceptable	640	4,140	3,355
True uncertain	176	117	640
	Predicted unacceptable	Predicted acceptable	Predicted uncertain

True unacceptable	470	241
True acceptable +uncertain	816	8,252
	Predicted unacceptable	Predicted acceptable +uncertain

Figure S4b. The one-step three-class 2D CNN approach

True unacceptable	418	93	200
True acceptable	72	7,252	811
True uncertain	93	343	497
	Predicted unacceptable	Predicted acceptable	Predicted uncertain

True unacceptable	418	293
True acceptable +uncertain	165	8,903
	Predicted unacceptable	Predicted acceptable +uncertain

Figure S4c. The two-step binary sequential random forest approach

True unacceptable	442	150	119
True acceptable	72	7,619	444
True uncertain	111	552	270
	Predicted unacceptable	Predicted acceptable	Predicted uncertain

True unacceptable	442	269
True acceptable +uncertain	183	8,885
	Predicted unacceptable	Predicted acceptable +uncertain

Figure S4d. The two-step binary sequential 2D CNN approach

Section S6. Source Codes

File S1a. Dataingestor.py (preprocess)

```
from genericpath import exists
import pandas as pd
import numpy as np
import pickle
import glob
import argparse
import os
from rsa import sign
from yaml import parse
import matplotlib
import matplotlib.pyplot as plt
from sklearn.preprocessing import MinMaxScaler
import heartpy
from scipy.interpolate import interp1d

class DataIngestor():

    def __init__(self, args) -> None:
        self.args=args
        self.fs=250
        self.datatype=args.datatype

    '''
    segments that are zero for atleast fs/2 samples
    '''
    def __zero_runs(self, a):
        # Create an array that is 1 where a is 0, and pad each end with an extra 0.
        iszero = np.concatenate(([0], np.equal(a, 0).view(np.int8), [0]))
        absdiff = np.abs(np.diff(iszero))
        # Runs start and end where absdiff is 1.
        ranges = np.where(absdiff == 1)[0].reshape(-1, 2)
        rangesdiff=np.diff(ranges).flatten()
        if(np.any(rangesdiff>=25)):
            return True
        return False

    '''
    segments that are constant for atleast fs/2 samples
    '''
    def __constant_runs(self, a):
        runs=np.arange(0,len(a),25)
        for i in runs:
            sample_run=a[i:i+25]
            if(np.all(sample_run==sample_run[0])):
                return True
        return False

    '''
    Interpolate signal
    '''
    def __interpolate_signal(self, signal):
        idx=np.nonzero(signal)[0]
        idxdiffs=np.diff(idx)
        if(len(idx)==len(signal)):
            return signal

        y=np.arange(len(signal))
        interp=interp1d(y[idx],signal[idx], fill_value='extrapolate')
        x=np.arange(len(y))
        signal_new=interp(x)
```

```
        return signal_new

    '''
    1. Read valid annotations written out by ingestannotations()
    2. Load npy segments inside datadir
    3. Ignore segments that are all zeros or constants
    4. If segment has zero or constant values for less than fs/2 samples, interpolate
    4. Write segments and signals as csv and pickle

    '''
    def remove_blank_segments(self):
        segments=glob.glob(self.args.datadir+'/*')
        signals=[]
        inds_to_delete=[]
        inds_to_keep=[]
        for sigind,f in zip(np.arange(len(segments)),segments):
            ecg_signal=np.load(f)[:,self.datatype]
            runs=np.arange(0,ecg_signal.shape[0],10)
            signal_agg=np.zeros((500))
            for i,j in zip(runs,np.arange(len(runs))):
                signal_agg[j]=np.mean(ecg_signal[i:i+10])
            if(np.all(signal_agg==0) or np.all(signal_agg==signal_agg[0])):
                # blank segment
                inds_to_delete.append(sigind)
                continue;
            if(self.__zero_runs(signal_agg) or self.__constant_runs(signal_agg)):
                inds_to_keep.append(sigind)
                signals.append(signal_agg)
            else:
                if(~(signal_agg==self.__interpolate_signal(signal_agg)).all()):
                    signal_agg=self.__interpolate_signal(signal_agg)

                inds_to_keep.append(sigind)
                signals.append(signal_agg)

        if inds_to_keep:
            segments_keep=[segments[i] for i in inds_to_keep ]
            pd.DataFrame(segments_keep).to_csv(os.path.join(args.saveLocation, 'segments_notblank.csv'), index=False, header=False)
        if inds_to_delete:
            excludedsegments=[]
            excludedsegments=[segments[i] for i in inds_to_delete]
            pd.DataFrame(excludedsegments).to_csv(os.path.join(args.saveLocation, 'excludedsegments_blank.csv'), index=False,
header=False)

        signals=np.asarray(signals)

        with open(os.path.join(args.saveLocation, 'signals__notblank.pkl'),'wb') as f:
            pickle.dump(signals, f)

    def __scale_minmax(signals):
        scaler=MinMaxScaler((0,1))
        return scaler.fit_transform(signals)

    def __remove_baseline_wander(self, signals):
        return heartpy.remove_baseline_wander(signals, sample_rate=self.fs)

    '''
    1. Read in signals written out by remove_blank_segments
    2. Convert signals to images for use in Resnets
    3. CAUTION: Depending on size of dataset, may take long time

    '''
    def __make_images(self, signals):

        matplotlib.use('Agg')
        os.makedirs(os.path.join(args.saveLocation, 'images'), exist_ok=True)
```

```
        for sig, e in zip(signals, np.arange(len(signals))):
            plt.figure()
            plt.plot(sig)
            plt.axis('off')
            plt.savefig(os.path.join(args.saveLocation, str(args.datatype)) + str(e) + ".png", bbox_inches='tight', pad_inches=0,    dpi=96,
figsize=(224,224))
            plt.cla()

'''
COMMAND LINE ARGS:
datadir - Directory where your data is present (as npy files)
saveLocation - path to store preprocessed signals and other csv files, must be different from datadir
datatype - 0 if ECG (default), 1 if ABP, 2 if ABP_MEAN (npy files in datadir will contain these tracks in said order)
'''
if __name__ == "__main__":

    parser=argparse.ArgumentParser(description='parse args to ingest ecg signals')
    parser.add_argument('--datadir', type=str, default='')
    parser.add_argument('--saveLocation', type=str, default='')
    parser.add_argument('--datatype', type=int, default=0)
    args=parser.parse_args()

    if(os.path.exists(args.datadir) and os.path.exists(args.saveLocation)):
        try:
            ingestor=DataIngestor(args)
            ingestor.remove_blank_segments()
        except Exception as e:
            print(e)

    else:
        print('Run program with --datadir --saveLocation --datatype argument params')
```

File S1b. FeatureExtractor.py (feature extraction codes for random forest model)

```
class FeatureExtractor():

    def __init__(self, args) -> None:

        self.args=args
        self.fs=250
        self.cutoff=[0.5,8]
        self.window_size=0.9
        self.N=1000
        self.n=numpy.arange(self.N)
        self.T=self.fs/self.N
        self.freq=self.n/self.T
        self.signals=[]
        self.featureset=[]

    def build_feature_matrix(self):
        self.signals=pandas.read_pickle(self.args.datadir)
        for signal in self.signals:
            self.featureset.append(self.__build_feature_set(signal))
        self.featureset=numpy.asarray(self.featureset)
        if self.args.datatype==0:
            signaltype='ecg'
        if self.args.datatype==1:
            signaltype='ppg'
        if self.args.datatype==2:
            signaltype='abp'

        numpy.save(self.args.saveLocation+'/features' + '-' + signaltype + '.npy', self.featureset)
```

```
    def __build_feature_set(self, signal):

        filtered_signal=self.__filter_signal(signal)
        filtered_signal_neg=self.__filter_signal(-signal)
        seg1=filtered_signal[:1000]
        seg2=filtered_signal[1000:2000]
        seg3=filtered_signal[2000:3000]
        seg4=filtered_signal[3000:4000]
        seg5=filtered_signal[4000:]
        invseg1=filtered_signal_neg[:1000]
        invseg2=filtered_signal_neg[1000:2000]
        invseg3=filtered_signal_neg[2000:3000]
        invseg4=filtered_signal_neg[3000:4000]
        invseg5=filtered_signal_neg[4000:]
        peaks,peakinds=self.__detectpeaks(filtered_signal)
        peaksinv,peakindsinv=self.__detectpeaks(filtered_signal_neg)
        peaks_z=zscore(peaks)
        peaks_zinv=zscore(peaksinv)
        sigfft=[self.__fft(seg1),self.__fft(seg2),self.__fft(seg3),self.__fft(seg4),self.__fft(seg5)]
        sigfftinv=[self.__fft(invseg1),self.__fft(invseg2),self.__fft(invseg3),self.__fft(invseg4),self.__fft(invseg5)]

se=[self.__spectralentropy(seg1),self.__spectralentropy(seg2),self.__spectralentropy(seg3),self.__spectralentropy(seg4),self.__spectralentropy(seg5
)]
        autocorr=[self.__autocorr(seg1),self.__autocorr(seg2),self.__autocorr(seg3),self.__autocorr(seg4),self.__autocorr(seg5)]
        autocorrinv=[self.__autocorr(invseg1),self.__autocorr(invseg2),self.__autocorr(invseg3),self.__autocorr(invseg4),self.__autocorr(invseg5)]

he=[self.__hurstexponent(seg1)[0],self.__hurstexponent(seg2)[0],self.__hurstexponent(seg3)[0],self.__hurstexponent(seg4)[0],self.__hurstexponent(
seg5)[0]]

heinv=[self.__hurstexponent(invseg1)[0],self.__hurstexponent(invseg2)[0],self.__hurstexponent(invseg3)[0],self.__hurstexponent(invseg4)[0],self.__h
urstexponent(invseg5)[0]]
        skeww=[self.__skew(seg1),self.__skew(seg2),self.__skew(seg3),self.__skew(seg4),self.__skew(seg5)]
        skewwinv=[self.__skew(invseg1),self.__skew(invseg2),self.__skew(invseg3),self.__skew(invseg4),self.__skew(invseg5)]
        kurtosiss=[self.__kurtosis(seg1),self.__kurtosis(seg2),self.__kurtosis(seg3),self.__kurtosis(seg4),self.__kurtosis(seg5)]

slopes=[self.__computeslope(seg1),self.__computeslope(seg2),self.__computeslope(seg3),self.__computeslope(seg4),self.__computeslope(seg5)]
        z=[self.__stft(seg1),self.__stft(seg2),self.__stft(seg3),self.__stft(seg4),self.__stft(seg5)]
        zinv=[self.__stft(invseg1),self.__stft(invseg2),self.__stft(invseg3),self.__stft(invseg4),self.__stft(invseg5)]
        fd=[self.__gradient(seg1), self.__gradient(seg2), self.__gradient(seg3), self.__gradient(seg4), self.__gradient(seg5)]
        fdinv=[self.__gradient(invseg1), self.__gradient(invseg2), self.__gradient(invseg3), self.__gradient(invseg4), self.__gradient(invseg5)]
        dtww=[]
        crosscorr=[]
        dtwwinv=[]
        crosscorrinv=[]
        for sig1 in [seg1,seg2,seg3,seg4,seg5]:
            for sig2 in [seg1,seg2,seg3,seg4,seg5]:
                if(~(sig1==sig2).all()):
                    dtww.append(self.__dynamictimewarping(sig1,sig2))
                    crosscorr.append(self.__crosscorr(sig1,sig2))
        for sig1 in [invseg1,invseg2,invseg3,invseg4,invseg5]:
            for sig2 in [invseg1,invseg2,invseg3,invseg4,invseg5]:
                if(~(sig1==sig2).all()):
                    dtwwinv.append(self.__dynamictimewarping(sig1,sig2))
                    crosscorrinv.append(self.__crosscorr(sig1,sig2))

        features=[self.__max(peaks_z), self.__min(peaks_z),
                  self.__max(peaks_zinv), self.__min(peaks_zinv),
                    self.__stdd(peaks), self.__stdd(peaksinv),
                    self.__stdd(peakinds), self.__stdd(peakindsinv),
                    self.__mean(z), self.__stdd(z),
                    self.__mean(zinv), self.__stdd(zinv),
                    self.__mean(slopes),self.__stdd(slopes),
                    self.__stdd(se),
                    self.__stdd(fd),
                    self.__stdd(fdinv),
                    self.__stdd(numpy.max(sigfft)), self.__stdd(numpy.max(sigfftinv)),
                    self.__mean(autocorr), self.__stdd(autocorr),
```

```
                    self.__mean(autocorrinv), self.__stdd(autocorrinv),
                    self.__mean(crosscorr), self.__stdd(crosscorr),
                    self.__mean(crosscorrinv), self.__stdd(crosscorrinv),
                    self.__stdd(skeww), self.__mean(skeww),
                    self.__stdd(skewwinv),self.__mean(skewwinv),
                    self.__stdd(kurtosiss), self.__mean(kurtosiss),
                    self.__mean(dtww), self.__stdd(dtww),
                    self.__mean(dtwwinv), self.__stdd(dtwwinv),
                    self.__stdd(peakinds), self.__stdd(peakindsinv),
                    self.__mean(he), self.__stdd(he),
                    self.__mean(heinv), self.__stdd(heinv)
                ]
        return numpy.asarray(features)

    def __mean(self, feature):
        return numpy.mean(feature)

    def __stdd(self, feature):
        return numpy.std(feature)

    def __max(self, feature):
        return numpy.max(feature)

    def __min(self, feature):
        return numpy.min(feature)

    def __skew(self, filt_signal):
        return skew(filt_signal)

    def __kurtosis(self, filt_signal):
        return kurtosis(filt_signal)

    def __hurstexponent(self, filt_signal):
        return hurst.compute_Hc(filt_signal)

    def __gradient(self, filt_signal):
        return numpy.gradient(filt_signal)

    def __autocorr(self, filt_signal):
        return sm.tsa.acf(filt_signal, nlags=1)[1]

    def __fft(self, filt_signal):
        return numpy.abs(fft(filt_signal))

    def __spectralentropy(self, filt_signal):
        return entropy_spectral(filt_signal)[0]

    def __computeslope(self, filt_signal):
        return linregress(numpy.arange(1000), filt_signal)[0]

    def __stft(self, filt_signal):
        f,t,z=stft(filt_signal, fs=self.fs, nperseg=100)
        return numpy.abs(z)

    def __dynamictimewarping(self, filt_signal1, filt_signal2):
        return dtw.distance_fast(filt_signal1, filt_signal2, use_pruning=True)

    def __crosscorr(self, filt_signal1, filt_signal2):
        return correlate(filt_signal1, filt_signal2).mean()

    def __filter_signal(self, signal):
        return heartpy.filtering.filter_signal(signal, sample_rate=self.fs, cutoff=self.cutoff, filtertype='bandpass')

    def __detectpeaks(self, filt_signal):
        rol_mean = heartpy.datautils.rolling_mean(filt_signal, windowsize = self.window_size, sample_rate = self.fs)
        wd=heartpy.peakdetection.detect_peaks(filt_signal, rol_mean=rol_mean, sample_rate=self.fs, ma_perc=20)
        peakinds=numpy.asarray(wd['peaklist'])
```

```
        peaks=numpy.asarray(wd['ybeat'])
        return peakinds,peaks

'''
COMMAND LINE ARGS:
datadir - preprocessed data path
saveLocation - path to store features
'''

if __name__ == "__main__":

    parser=argparse.ArgumentParser(description='parse args to ingest ecg signals')
    parser.add_argument('--datadir', type=str, default='')
    parser.add_argument('--saveLocation', type=str, default='')
    parser.add_argument('--datatype', type=str, default=0)

    args=parser.parse_args()

    if(os.path.exists(args.datadir) and os.path.exists(args.saveLocation)):
        try:
            featureextractor=FeatureExtractor(args)
            featureextractor.build_feature_matrix()
        except Exception as e:
            print(e)

    else:
        print('Run program with --datadir --saveLocation argument params')
```

File S1c. Codes for training random forest model for differentiation between *acceptable* and *unacceptable*

```
#!/usr/bin/python
# -*- coding: utf-8 -*-
import joblib
from sklearn.feature_selection import SelectKBest, mutual_info_classif
from sklearn.model_selection import train_test_split
from sklearn.ensemble import RandomForestClassifier
from sklearn.metrics import confusion_matrix, accuracy_score, \
    precision_recall_fscore_support

import numpy, random

trainfeats = \
    numpy.load('random-forests/exclude-vs-include-features-training.npy'
               )
trainlabels = \
    numpy.load('random-forests/exclude-include-labels-training.npy')

excinds = numpy.where(trainlabels == 0)[0]
incinds = numpy.where(trainlabels == 1)[0]
numpy.random.shuffle(incinds)
numpy.random.shuffle(excinds)

batches = []
rfc_inc_exc = []
num_trees = 100
precisions = []
recalls = []
models = []
kbestf = []
```

```
for trial in numpy.arange(20):
    inds = random.choices(incinds, k=600)
    include_batch = trainfeats[incinds[inds], :]
    include_batch_labels = trainlabels[incinds[inds]]
    train_batch = numpy.vstack([trainfeats[excinds], include_batch])
    train_batch_labels = numpy.hstack([trainlabels[excinds],
            include_batch_labels])
    train_data = numpy.c_[train_batch, train_batch_labels]
    numpy.random.shuffle(train_data)
    train_batch = train_data[:, :-1]
    train_batch_labels = train_data[:, -1]
    kbest = SelectKBest(mutual_info_classif, k=43)
    Xy_inc_exc_kbest = \
        kbest.fit_transform(numpy.nan_to_num(train_batch),
                            train_batch_labels)
    (Xtrain_inc_exc, Xtest_inc_exc, ytrain_inc_exc, ytest_inc_exc) = \
        train_test_split(Xy_inc_exc_kbest, train_batch_labels,
                         test_size=0.2, random_state=2022, shuffle=True)
    if rfc_inc_exc == []:
        rfc_inc_exc = RandomForestClassifier(n_estimators=num_trees,
                random_state=2022, oob_score=True, warm_start=True)
        rfc_inc_exc = rfc_inc_exc.fit(Xtrain_inc_exc, ytrain_inc_exc)
    else:
        rfc_inc_exc.n_estimators = num_trees + 100
        rfc_inc_exc = rfc_inc_exc.fit(Xtrain_inc_exc, ytrain_inc_exc)
    preds_inc_exc = rfc_inc_exc.predict(Xtest_inc_exc)
    (prec, rec, fsc, acc) = \
        precision_recall_fscore_support(ytest_inc_exc, preds_inc_exc)
    models.append(rfc_inc_exc)
    kbestf.append(kbest.get_feature_names_out())
    precisions.append(prec)
    recalls.append(rec)

metrics = numpy.c_[precisions, recalls]
threshold = 0.9
rows = numpy.where((metrics > threshold).all(1))[0]
modelsselected_incexc = []
kbestindss_incexc = []
joblib.dump(models[0], 'random-forests/rfc-2-step-inc-vs-exc.joblib')
kbestfeats = numpy.asarray(kbestf)[0]
kbestinds = []
for f in kbestfeats:
    kbestinds.append(numpy.int(''.join(list(f)[1:])))
kbestinds = numpy.asarray(kbestinds)
print ('kbestinds:', kbestinds)
numpy.save('random-forests/kbest-2-step-rfc-inc-vs-exc.npy', kbestinds)
```

File S1d. Codes for training of 2D CNN (binary classifier for *unacceptable* vs *uncertain*)

```
from scipy.signal import stft
import scipy.fft as fft
import pickle
import numpy as np
import pandas as pd
import matplotlib.pyplot as plt
from sklearn.model_selection import train_test_split
import tensorflow as tf
from tensorflow.keras.layers import Flatten, Dense, Conv2D, MaxPool2D, AveragePooling2D,Dropout,BatchNormalization
from tensorflow.keras.utils import to_categorical
import import_ipynb
import metrics

signals='C:/Users/SNUBH/….pkl'
labels='C:/Users/SNUBH/….pkl'
```

```
preprocessed_signals=pd.read_pickle(signals)
labels=pd.read_pickle(labels)


include_inds=np.where(labels==1)[0]

# Drop all acceptable data
preprocessed_signals_without_include=np.delete(preprocessed_signals, include_inds, axis=0)
labels_without_include=np.delete(labels, include_inds)
print(preprocessed_signals_without_include.shape)

labels_without_include[labels_without_include==0.5]=1


# train with an equal number of samples
exclude_inds=np.where(labels_without_include==0)[0]
np.random.shuffle(exclude_inds)
exclude_signals=preprocessed_signals_without_include[exclude_inds[:750],:]
exclude_signals_reserved=preprocessed_signals_without_include[np.setdiff1d(exclude_inds,select),:]

uncertain_inds=np.where(labels_without_include==1)[0]
uncertain_signals=preprocessed_signals_without_include[uncertain_inds[:550],:]

exclude_signals_aug=-exclude_signals
uncertain_signals_aug=-uncertain_signals


exclude_signals_aug=np.vstack([exclude_signals,exclude_signals_aug])
uncertain_signals_aug=np.vstack([uncertain_signals,uncertain_signals_aug])

# Create sequential model1
import import_ipynb
import metrics
model1=[]
model1 = tf.keras.models.Sequential(name="simple_cnn_model1")


model1.add(Conv2D(filters=100, kernel_size=8, padding='same', input_shape
                        = (80,100,1)))
model1.add(tf.keras.layers.LeakyReLU(alpha=0.002))
model1.add(AveragePooling2D(pool_size=(10,10), strides=(3,3), padding='same'))

model1.add(Conv2D(filters=75, kernel_size=6, padding='same'))
model1.add(tf.keras.layers.LeakyReLU(alpha=0.002))
model1.add(AveragePooling2D(pool_size=(5,5), strides=(3,3), padding='same'))

model1.add(Conv2D(filters=50, kernel_size=6, padding='same'))
model1.add(tf.keras.layers.LeakyReLU(alpha=0.002))
model1.add(AveragePooling2D(pool_size=(5,5), strides=(3,3), padding='same'))

model1.add(Conv2D(filters=25, kernel_size=4, padding='same'))
model1.add(tf.keras.layers.LeakyReLU(alpha=0.002))
model1.add(AveragePooling2D(pool_size=(3,3), strides=(3,3), padding='same'))

model1.add(Dropout(0.5))

model1.add(Flatten())

#Add a dense layer with 128 neurons
model1.add(Dense(units = 128, use_bias=True,kernel_initializer=tf.keras.initializers.GlorotNormal(), bias_initializer='zeros'))
model1.add(Dense(units = 64, use_bias=True,kernel_initializer=tf.keras.initializers.GlorotNormal(), bias_initializer='zeros'))
model1.add(Dense(units = 32, use_bias=True,kernel_initializer=tf.keras.initializers.GlorotNormal(), bias_initializer='zeros'))
model1.add(Dense(units = 16, use_bias=True,kernel_initializer=tf.keras.initializers.GlorotNormal(), bias_initializer='zeros'))

model1.add(Dense(activation='softmax', units = 2, use_bias=True,kernel_initializer=tf.keras.initializers.GlorotNormal(), bias_initializer='zeros'))
```

```
model1.add(tf.keras.layers.LeakyReLU(alpha=0.002))
model1.add(Flatten())


callback=tf.keras.callbacks.EarlyStopping(
    monitor='val_loss',
    min_delta=0,
    patience=3,
    verbose=0,
    mode='auto',
    baseline=None,
    restore_best_weights=True
)
model1.compile(optimizer= tf.keras.optimizers.Adam(learning_rate=0.002), loss = 'binary_crossentropy', metrics=metrics.METRICS)
trials=20
from sklearn.metrics import confusion_matrix,precision_recall_fscore_support
import random

for trial in np.arange(trials):
    # random shuffle, split train-test
    exclude_signals_ss=random.choices(exclude_signals_aug, k=1100)
    new_data=np.vstack([exclude_signals_ss, uncertain_signals_aug])
    new_labels=np.append(np.zeros(len(exclude_signals_ss)), np.ones(len(uncertain_signals_aug)))
    xtrain,xtest,ytrain,ytest=train_test_split(new_data, new_labels, test_size=0.03, random_state=0, shuffle=True)
    print(xtrain.shape, ytrain.shape)
    print(xtest.shape, ytest.shape)
    ytrain_cat = to_categorical(ytrain, 2)
    ytest_cat = to_categorical(ytest, 2)

    reshaped_xtrain=np.zeros((xtrain.shape[0],800,10))
    reshaped_xtest=np.zeros((xtest.shape[0],800,10))

    print(reshaped_xtrain.shape)
    for i in np.arange(xtrain.shape[0]):
        for sample,j in zip(np.arange(0,5000,5), np.arange(800)):
            reshaped_xtrain[i,j,:]=xtrain[i,sample:sample+10]
    reshaped_xtrain = reshaped_xtrain.reshape(reshaped_xtrain.shape[0], 80,
                                              100,
                                              1)

    for i in np.arange(xtest.shape[0]):
        for sample,j in zip(np.arange(0,5000,5), np.arange(800)):
            reshaped_xtest[i,j,:]=xtest[i,sample:sample+10]
    reshaped_xtest = reshaped_xtest.reshape(reshaped_xtest.shape[0], 80,
                                            100,
                                            1)

    cnn_model1_history = model1.fit(reshaped_xtrain, ytrain_cat, epochs=50, batch_size = 25,
                                    validation_split=0.1, callbacks=callback)
    pred=model1.predict(reshaped_xtest)
    pred=pd.DataFrame(pred)
    pred=pred.idxmax(axis=1)
    print(confusion_matrix(ytest,pred))
    model1.optimizer.learning_rate=model1.optimizer.learning_rate*0.01

    import statistics
    print(statistics.mean(cnn_model1_history.history['loss']), statistics.mean(cnn_model1_history.history['val_loss']))
```
